# Postprandial Glycaemic Responses of Dried Fruit-Containing Meals in Healthy Adults: Results from a Randomised Trial

**DOI:** 10.3390/nu10060694

**Published:** 2018-05-30

**Authors:** Ruixin Zhu, Zhihong Fan, Yang Dong, Manman Liu, Linlin Wang, Haikun Pan

**Affiliations:** 1 Beijing Advanced Innovation Centre for Food Nutrition and Human Health, China Agricultural University, Beijing 100083, China; zhuruixin07@126.com (R.Z.); feidi@sina.com (Y.D.); lynn9523@126.com (L.W.); m15201424460_3@163.com (H.P.); 2 Department of Food Science and Engineering, College of Biological Science and Technology, Beijing Forestry University, Beijing 100083, China; manman_Liu@bjfu.edu.cn

**Keywords:** glycaemic responses, mixed diets, dried fruits, almonds, dried jujubes

## Abstract

The aim of this study was to explore the glycaemic response (GR) patterns of four dried fruits (DF) and the mixed meals containing dried fruits, rice and almonds. Dried apples (DApp), dried jujubes (DJ), raisins (Ra) and dried apricots (DApr) were tested in eleven healthy subjects in random order. Test meals included the following 3 groups: (1) dried fruits containing 50 g available carbohydrates; (2) mixed meals consisting of DF and rice (DF + R), each contributing 25 g available carbohydrates; (3) Group (2) supplemented with 30 g almonds (DF + R + A). The postprandial GR and other characteristics in 240 min were investigated. The GI values of 4 DFs were 43 for DApp, 55 for DJ, 56 for both Ra and DApr. The DApp displayed the smallest amplitude of glycaemic excursion within 240 min (MAGE_0–240_). Compared with rice, the DApp + R meal elicited a significantly reduced GR and a smaller MAGE_0–240_ (GI 81 vs. 65). With the addition of almonds, the GIs and MAGE_0–240_ decreased significantly in all DF + A + R combinations except DApp + R + A. The ratio of total fructose/glucose contents of test meals were negatively correlated to GIs. Dried fruits and nuts may have the potential to mitigate the postprandial GR when jointly introduced into glycaemic management diets.

## 1. Introduction

Nuts and dried fruit (DF) are traditional dietary components in many countries, and have been included in healthy diet patterns, such as the Dietary Approaches to Stop Hypertension (DASH) diet [1,2] and the Mediterranean diet (MedDiet) [3], as they are a good source of dietary fibre, potassium, magnesium and antioxidants such as polyphenols [4,5]. The consumption of nuts was encouraged by the American Diabetes Association (ADA) in their nutrition recommendations [6] as their benefits in cardiovascular disease prevention [7] and glycaemic control [8] had been well established. However, in contrast to nuts, DF may not be as easily accepted as a group of healthy food by the diabetic and people of impaired glucose tolerance because these dried fruits are considered to be high in sugar.

Recent studies showed that DFs such as raisins were medium-to-low glycaemic index (GI) food [9,10,11], and could elicit favourable physiologic responses in terms of insulin secretion and appetite modulating hormones [12]. Raisins were also reported for their benefits to cardiovascular disease risk factors including inflammation status, vascular endothelial functions, lipoprotein profiles [11] and blood pressure control, when consumed as a substitution of highly processed snacks [13]. Dried plums and dried apples showed effects on lowering serum hydroperoxides, C-reaction protein and low-density lipoprotein cholesterol (LDL-c) levels [14]. Dried jujubes were traditionally regarded as healthy food and a source of phytochemicals in East Asian countries [15,16]. Based on these previous studies, it is reasonable to investigate the possibility of incorporating DF into a nutritious, low-GI and high-fibre diet, which has been suggested to be beneficial to the people at a high risk of diabetes.

Given the fact that DFs are high in sugar, in order to avoid a shift of the energy balance and macronutrient distribution, the consumption of DF should be considered to be a replacement of other foods rich in sugar or starch. In fact, DFs are traditionally consumed as an ingredient of carbohydrate-based food, such as bread, steamed bread, other baked foods and rice foods, in many cultures. However, the glycaemic response of mixed meal consisting of DFs and starch food, on the basis of the same amount of available carbohydrate, is rarely reported.

Previous studies showed that adding nuts such as almonds into white bread meals could suppress the postprandial glycaemic response [17]. However, the effect of the combination of nuts and DF on blood glucose after a carbohydrate-based meal has not yet been investigated.

The aim of this study was to investigate the effect of several DFs on acute postprandial glycaemia in 240 min, either consumed alone or included in mixed meals of the same level of carbohydrate intake. We also examined the synergic contribution of DFs and nuts on the glycaemic excursion pattern when co-ingested with high-GI white rice.

## 2. Experimental Methods

### 2.1. Subjects

Healthy young volunteers aged between 18 and 25 were recruited through advertisements on the university bulletin board and BBS online. The questionnaires given to subjects contained the following criteria: (1) non-smoker; (2) non-drinker; (3) free from food allergies; (4) stable weight in the past three months; (5) regularly eating three meals and not breakfast skipper; (6) not on diet to gain or to lose weight; (7) not on medication in the past six months; (8) no metabolic disease or impaired glucose tolerance; (9) not in pregnancy. The sample size was calculated using the PASS 13 *Power Analysis and Sample Size* software (NCSS, Kaysville, UT, USA). It found that the test would have 80% power to examine a difference (*p* < 0.05) with six subjects in iAUC of 145.5 mmol/L·2 h, assuming that the standard deviation (SD) is lower than 104.4 mmol/L·2 h. These calculations were based on a 2-treatment, randomised, crossover study where they observed a 46.2% reduction (145.5 mmol/L·2 h) in iAUC for raisins compared with the glucose control [9]. Eleven potential subjects who met these criteria were invited to the laboratory and involved in additional assessments—an oral glucose tolerance test (OGTT) and GR to rice, both were tested twice. The informed consent forms were signed by all eleven eligible individuals. The study was conducted in accordance with the Declaration of Helsinki and carried out at the College of Food Science and Nutritional Engineering, China Agricultural University. The study protocol was approved by the Ethics Committee of China Agricultural University (ethics number 2015027).

### 2.2. Study Design

The study used a randomised crossover design where participants consumed test meals in a randomised order on seventeen separate mornings. Subjects were enrolled to this study using a single allocate ratio. The wash-out period was one week between each test session. Twenty-four hours before each trial day, the subjects were asked to refrain from drinking coffee or alcoholic beverages, excessive eating, staying up late and strenuous exercise. Participants were instructed to consume the test meal in 15 min. Subjects were served 200 mL of water in room temperature between 90 and 120 min. They were provided with books, magazines and Wi-Fi, and were suggested to stay seated during the test session but asked neither to consume food that was not related to the study nor to discuss the test meal. The glucose reference and the pure rice meal samples were tested twice respectively in order to assess the reliability of the study procedure.

### 2.3. Pretreatment of Test Dried Fruits

Raisins (*Vitis vinifera* Linn.), dried apples (*Malus pumila* Mill.), dried jujubes (*Ziziphus jujuba* Mill.), dried apricots (*Armeniaca vulgaris* Lam.), and almonds (*Armeniaca vulgaris* Lam.) were selected as raw materials. Dried fruit portions which contained 50.0 g of available carbohydrates were put in the Tupperware^®^ containers and stored in a refrigerator at 4 °C. Containers were fetched out 12 h before the experiment to enable an equilibration to the room temperature.

### 2.4. Preparation of Test Meals

The test meals included three groups: (1) four DFs including raisins (Ra), dried apples (DApp), dried jujubes (DJ) and dried apricots (DApr), each containing 50.0 g of glucose; (2) mixed meals of DF and rice (DF + R), each contributing 25.0 g of available carbohydrates, including mixed meals of dried apples and rice (DApp + R), mixed meals of raisins and rice (Ra + R), mixed meals of dried jujubes and rice (DJ + R), and mixed meals of dried apricots and rice (DApr + R); (3) mixed meals supplemented with 30.0 g of almonds as well as the above combination of DF and rice, including the mixed meals of raisin, almonds and rice (Ra + A + R), mixed meals of almonds, dried apples and rice (DApp + A + R), mixed meals of dried jujubes, almonds and rice (DJ + A + R), and mixed meals of dried apricots, almonds and rice (DApr + A + R) as well as mixed meals of almonds and rice (A + R). In the morning of test days, each rice portion (66.1 g of *japonica* rice and 99.0 g of water) was steamed for 40 min in a plastic crisper, and served to subjects at 40 °C. The glucose (50.0 g of Glucolin™ dissolved in 250 mL of water in room temperature) and rice containing 50 g of available carbohydrates were prepared as dual reference foods. The composition of the test meals is shown in Table 1.

### 2.5. Chemical Analysis of Test Meals

The composition of the test meals was analysed as follows. Fructose, glucose and sucrose were analysed using a high-performance liquid chromatography method with evaporative light scattering detection (HPLC-ELSD) [18]. Total acid contents were estimated by potentiometric titration [19]. Pectin were hydrolysed using the method of Ahmed and Labavitch [20]. The uronic acid contents were determined using a colorimetric method according to Filisetti-Cozzi and Carpita [21]. Total dietary fibre contents were examined gravimetrically according to AOAC 985.28 [22]. Total phenolic compound contents were determined with Folin–Ciocalteu reagents according to the method of Singleton et al. [23], and gallic acid was used as a standard phenolic compound. The analysis was carried out on a Phenomenex Luna 5u NH2 100 A column with isocratic elution of acetonitrile: water (82.5:17.5, *v*/*v*). The oxygen radical absorbing capacity (ORAC) were determined according to Ou et al. [24] using Trolox as a standard compound.

### 2.6. Blood Glucose Measurement

The glycaemic test protocol used was an adopted form that recommended by the Food and Agricultural Organization (FAO) and the World Health Organization (WHO). The subjects were asked to arrive at the laboratory at 8:00 a.m., and their fasting plasma glucose concentrations were tested after a 10-min rest. The test meals were provided by a person who was not involved in data analysis to the subjects at 8:15 a.m., and the finger prick blood samples were collected at 15, 30, 45, 60, 90, 120, 150, 180, 210 and 240 min just following the start of the test meal. The second drop of blood was used for testing to avoid possible plasma dilution. Plasma blood glucose concentrations were measured on an ONETOUCH^®^ Ultra^®^ (LifeScan Inc., Milpitas, CA, USA) glucometer using the glucose oxidase method. The glucose oxidase method is considered to perform similarly to a standardized method in terms of evaluating the blood glucose concentrations [25,26].

### 2.7. Statistical Analysis

The GR data were converted to the value of glucose changes from the baseline. The incremental areas under the curve of postprandial GRs (iAUC), the incremental peak of blood glucose concentrations, the maximum amplitudes of glucose excursion in 240 min (MAGE_0–240_), and the negative area under the curve (NAUC) [27] were calculated. The iAUCs in different periods were calculated using the trapezoidal method [28], ignoring the area beneath the baseline level. The GI was determined from the iAUC areas of each test meal and that of the glucose control. The statistical analysis was performed using the SPSS version 21.0 (SPSS Inc., Chicago, IL, USA) and the data were showed as the means (standard deviations, SD) or the means (standard errors, SE) where appropriate. The blood glucose test values between the meals were compared using a one-way analysis of variance ANOVA, and Tukey’s multiple was used to adjust for the multiple comparisons test. The criterion for statistical significance was a two-tailed *p* < 0.05. The correlation of data was determined using Pearson correlation analysis.

## 3. Results

### 3.1. Subject Characteristics

All of the eleven subjects completed the 17 blood glucose test sessions. Their baseline characteristics are shown in Table 2. 

### 3.2. Blood Glucose of Four Dried Fruits

**Figure 1:**
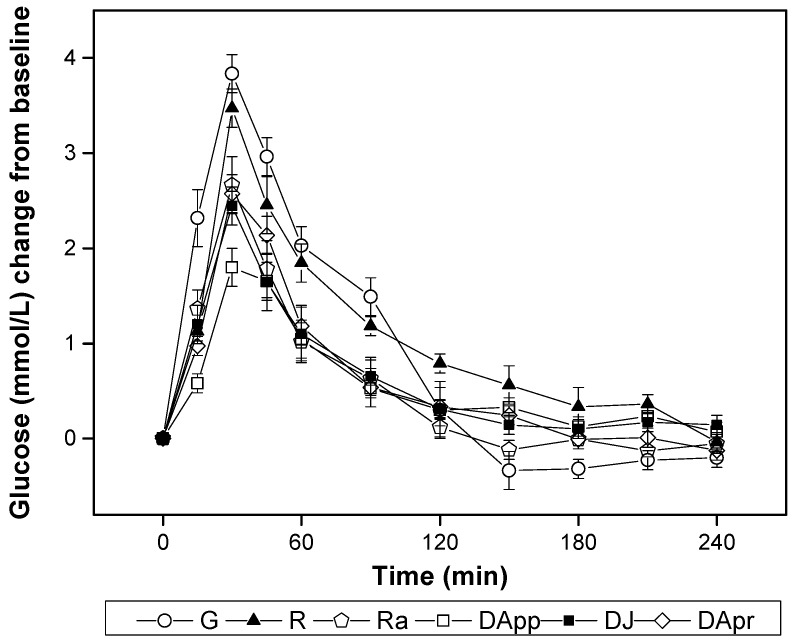
Postprandial plasma glucose changes (mean (SE)) in subjects (*n* = 11) after the consumption of test meals with glucose and rice as references (G, glucose; R, rice; Ra, raisins; DApp, dried apples; DJ, dried jujubes; DApr, dried apricots).

The curves of postprandial glucose response to four DFs are shown in Figure 1. The peak levels of glucose of all test samples attained at 30 min. Compared with other samples, the glucose level of DApp showed a significantly lower increment value (0.6 (SE 0.1), *p* = 0.020) at 15 min and (1.8 (SE 0.2), *p* = 0.027) 30 min. All DFs elicited significantly lower glucose increment values than those of the rice reference from 90 to 120 min (*p* < 0.05).

### 3.3. Blood Glucose of Mixed Meals of Dried Fruits and Rice

**Figure 2:**
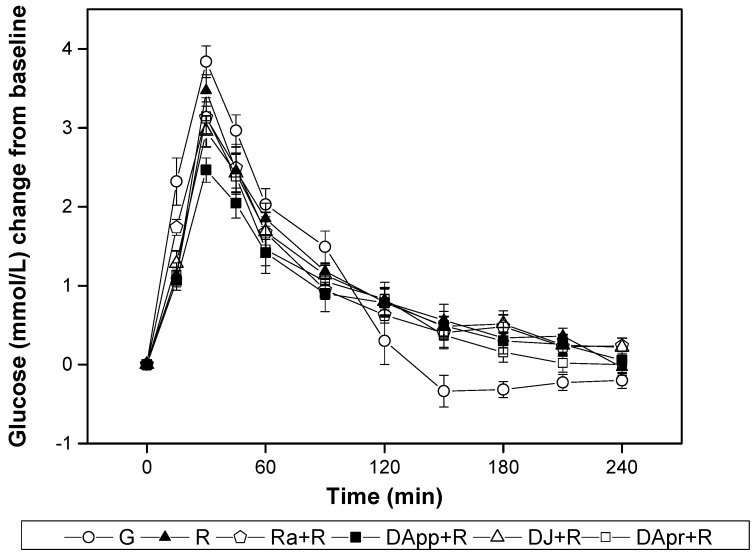
Postprandial plasma glucose changes (mean (SE)) in subjects (*n* = 11) after the consumption of test meals with glucose and rice as references (G, glucose; R, rice; Ra + R, mixed meal of raisins and rice; DApp + R, mixed meal of dried apples and rice; DJ + R, mixed meal of dried jujubes and rice; DApr + R, mixed meal of dried apricots and rice).

The glycaemic curves of 4 DFs containing meals (half of available carbohydrate from DFs) are shown in Figure 2. The peak level of blood glucose of all mixed meals attained at 30 min. Compared with the blood glucose changes of rice, the Ra + R meal elicited a significantly higher increment value (1.7 (SE 0.2), *p* = 0.021) at 15 min. The DApp + R meal showed a significantly lower increment value (2.0 (SE 0.2), *p* = 0.033) at 45 min, while DJ + R and Ra + R had significantly higher increment values (0.2 (SE 0.1), *p* = 0.045; 0.2 (SE 0.1), *p* = 0.040, respectively) at 240 min.

### 3.4. Blood Glucose of Mixed Meals of Dried Fruits, Almond and Rice

**Figure 3:**
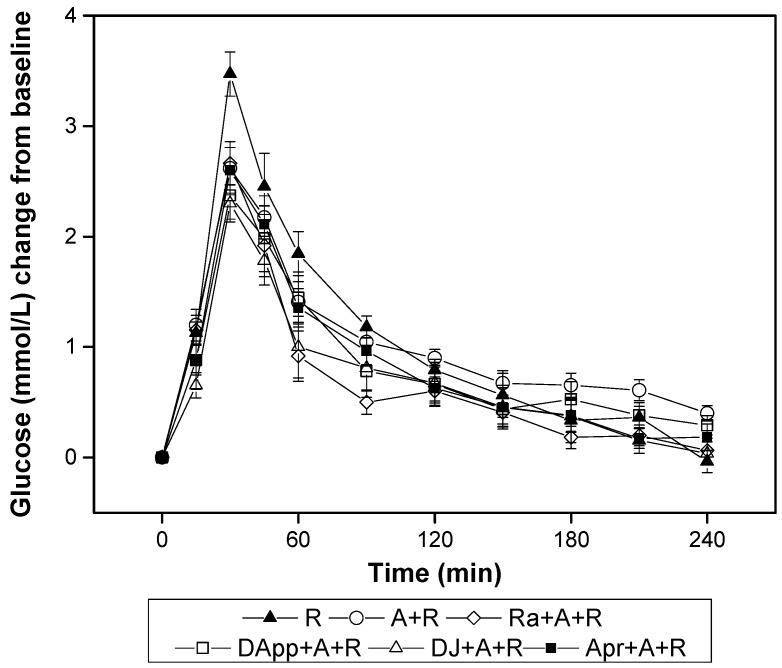
Postprandial plasma glucose changes (mean (SE)) in subjects (*n* = 11) after the consumption of test meals with glucose and rice as references (G, glucose; R, rice; A + R, mixed meal of almonds and rice; Ra + A + R, mixed meal of raisin, almonds and rice; DApp + A + R, mixed meal of almonds, dried apples and rice; DJ + A + R, mixed meal of dried jujubes, almonds and rice; DApr + A + R, mixed meal of dried apricots, almonds and rice.

The combination of almonds and DFs further mitigated the postprandial blood glucose responses of the mixed meals, especially at the 15 min point, as shown in Figure 3. The peak level of blood glucose of all mixed meals attained at 30 min. Among the four DF-almond mixed meals, only DJ + A + R showed a significantly lower increment value (0.7 (SE 0.2), *p* = 0.041) compared with rice (reference food) at 15 min. There were no differences between the four DF-almond mixed meals in other periods.

### 3.5. Postprandial Glycaemic Response Characteristics

As shown in Table 3, the peak values, the iAUC_0–60_s, iAUC_60–120_s, and iAUC_0–120_s of DApp, DJ and DApr were significantly smaller than those of the rice reference. The four DFs elicited significantly smaller glycaemic excursions during 240 min compared with the glucose reference, and among which the DApp and DJ had smaller MAGE_0–240_s compared with that of rice. It is worth noting that from 120 min onwards, the glucose level for the raisin and glucose reference dropped below the baseline, while the blood glucose levels of the DApp, DJ and rice remained constant, above the baseline and produced a significant smaller negative area under curve (NAUC) compared with those of Ra and glucose. The addition of both DApp and DJ caused a significantly reduction in peak values, iAUC_60–120_s and MAGE_0–240_s than rice alone, but only DApp + R produced the smallest iAUC_0–60_ and iAUC_60–120_. Ra + A + R and DJ + A + R had a significant reduction of blood glucose values than the Ra + R and DJ + R did during 0–60 and 60–120 min respectively. Interestingly, the addition of almonds resulted in significant drops in peak values, iAUC_0–120_s, MAGE_0–240_s and GIs in Ra + A + R and DJ + A + R, but not in DApp + A + R, compared with the corresponding mixed meals without almonds. 

The GI values of 4 DFs were all significantly lower than that of rice, but there were no differences between 4 DFs, and so did 4 dried fruit-rice mixed meals (Table 4). The GI values of DApp + A + R, DJ + A + R and Ra + A + R were significantly lower than those of A + R and DApr + A + R, which were already lower than the GI of rice. The GI values of DF with rice were significantly lower than those of DF or DF with rice and almonds, except for DApr + A + R.

### 3.6. Food Components Relevant to Blood Glucose Management

Sugar profiles and other components of dried fruits are shown in Table 5 and Table 6.

Correlation analysis in Table 7 indicated that the amount of total glucose (the combination of free glucose and the glucose unit in sucrose) in test meals had a significant positive correlation with the iAUC_0–60_, iAUC_0–120_ and NAUC. The ratio of the amount of total fructose and total glucose in test meals had a very strong negative correlation with the iAUC_0–60_, iAUC_0–120_ and peak value. No association was found between glycaemic characteristics and the content of the total carbohydrate, dietary fibre, pectin, organic acid or ORAC.

## 4. Discussion

The present study demonstrated that the adding of dried fruits to white rice meals, on the basis of isoenergetic exchange for other carbohydrates (the total amount of carbohydrate was kept constant at about 50 g), did not increase the GI of the mixed meals, despite of the considerate amount of simple carbohydrates including glucose and fructose. The joint effect of DF and nuts resulted in significant reduction of GI values as well as the glycaemic excursion in 240 min.

To our knowledge, this is the first report on GI value of dried jujubes. Additionally, the GI values of dried apples and dried apricots in the present study were higher than those in previous reports, which were 29 and 30 [29], respectively, while the GI of raisins (56) was comparable to the values for 64 [29], 49 [11] and 49–55 [9] in previous reports. The differences of GI values could be explained by the variety differencesof the DFs, methods used to determine GIs, e.g., the glucose oxidase method, the hexokinase method, the glucose dehydrogenase method, etc., as well as the physiological and ethnic differences of subjects. It was reported that the same sample might elicit a higher postprandial glucose response in Asian subjects compared with that in Western subjects [30].

Given the fact that the GI value of raisins in type 2 diabetes patients was lower than that determined in healthy subjects [31], it could possibly incur hypoglycaemic episodes after 120 min when ingested in a large amount. The dried apple and dried jujube, which demonstrated stable blood glucose levels through 120–240 min, might be regarded as better snack choice for people of impaired glucose control.

There may be multiple factors affecting the glycaemic properties of DFs, which include: (1) unavailable carbohydrates such as insoluble fibre, oligosaccharides and pectin [32,33]; (2) the amount and profile of sugar, i.e., the contents of glucose, fructose and sucrose [34,35]; (3) digestion enzyme inhibitors such as polyphenols [36]; (4) organic acid [37]; (5) the physical texture and chewy properties of the food [38].

Most of the abovementioned components were determined in this study. It was found that the glycaemic characteristics had no significant correlation with the contents of the total carbohydrate, dietary fibre, pectin, or organic acid. The dried apricot, which had the highest contents of total acid and pectin, and the raisins, which had the highest polyphenol contents and highest ORAC among the four dried fruits, failed to produce low GI values as the dried apple did. An analysis of 121 food GI tests showed that the dietary fibre content had no correlation with GI value [39]. Another study found that neither the carbohydrate content nor the dietary fibre content was the determinant of the GI value of potato varieties [40].

However, the sugar profile seemed to have an important impact on glycaemic responses of dried fruits. High intake of glucose component led to a rapid elevation of blood glucose within 60 min and a large range of fluctuation. The ratio of the amount of total fructose and total glucose in test meals had a very strong negative correlation with the iAUC_0–60_, iAUC_0–120_ and peak value. The low GI value of apples [29] and its benefit to type 2 diabetes prevention found in prospective cohort studies [41] may partly be explained by the fact that apples had a high content of total fructose and fructose/glucose ratio [4].

Although a large amount of fructose may incur an adverse metabolic impact [34], there is evidence that ‘catalytic’ doses of fructose from fruits could decrease the glycaemic response to high GI meals in human subjects without any unfavourable effects [42]. Small amounts of fructose have been shown to decrease the hepatic glucose production while accelerate the glycogen production [43], and thus, enhance hepatic glucose metabolism and result in better glycaemic control. A meta-analysis showed that isocaloric exchange of fructose for other carbohydrates could decrease the level of glycated blood proteins without affecting insulin in diabetes patients [34]. However, another meta-analysis of diet-intervention studies found that 26–293 g of fructose ingestion promoted the development of hepatic insulin resistance in non-diabetic subjects [44]. In the present study, the amount of total fructose in DApp + R, DJ + R and Ra + R diet was calculated to be 16.9 g, 12.9 g and 11.5 g, respectively, well below the threshold of 60 g/day [45]. Such a low dose of isocaloric fructose exposure (35–45 g dried fruit) is unlikely to incur any adverse effect on other aspects of metabolic control.

A previous study showed that as low as 30 g of almonds exerted acute post-prandial benefits when added to a high GI carbohydrate-based meal [17]. Preloading protein and fat prior to the ingestion of high carbohydrate could raise glucagon-like peptide 1 (GLP-1) levels and slow gastric empty rates, which contributes to reducing and delaying postprandial glycaemia [46]. However, in the present study, the addition of almond reduced the peak glucose concentrations and the iAUC of the meals without changing the time when peak values were attained.

It is worth noting that in the Ra + A + R meal, the addition of rice to raisins prevented the possible hypoglycaemia after 120 min which was seen in a pure raisin diet, while the incorporation of almonds effectively attenuated the hyperglycaemia at 15, 60 and 90 min seen in a pure rice or raisin meal. The combinations of almonds, dried fruit and rice could produce a smaller MAGE_0–240_ on a fixed amount of carbohydrate-based meal, while the minimised glycaemic excursion in the long term would be desirable for prevention of many complications associated with diabetes [47]. Thus, the mixture of DF and nuts could possibly be developed as a healthy snack, tea break food or preload food for people who need to monitor their blood glucose levels, either the hyperglycaemia or hypoglycaemia patients.

To our knowledge, this is the first report on the joint effect of DF and nuts on glycaemic responses in carbohydrate-based meals. In the present study, all test meals were based on almost equal amounts of carbohydrates, and well accepted by all the subjects. The chemical components including dietary fibre, the pectin content, the polyphenol content, the sugar profile and the organic acid, which were possible contributors to postprandial glucose control, were determined before the blood glucose tests. The correlation analysis between food components and glycaemic characteristics of test meals led to a better explanation of research results.

There are several limitations of the present study. First, the study was carried out on healthy subjects. The results need to be confirmed in people of impaired glucose control in further studies. Second, this is an acute feeding study, which could not be directly extrapolated to a sustainable glycaemic mitigating effect in a longer period of time. Third, the insulinaemic response and the gastrointestinal hormones were not determined in this study, while they would provide a more in-depth glycaemic control mechanism of the mixed diet. Finally, we used a ONETOUCH^®^ Ultra^®^ blood glucose glucometer as a device to measure blood glucose. While a study found this small glucometer met ISO 15197 [48], the evidence of accuracy of it is not strong. So, a comparison ofthe glycaemic responses using a ONETOUCH^®^ Ultra^®^ glucometer and a standard laboratory glucose analyser should be involved in the further study.

In conclusion, the present study demonstrated that dried fruits, including dried apples, dried jujubes, dried apricots and raisins, are medium or low GI foods, which would not elicit the excess rise in blood glucose concentrations when consumed as a substitute of high GI carbohydrate-based food. Moderate amounts of sugar, especially the fructose from dried fruit, may help postprandial glycaemic control. Taking the nutrient profile and antioxidants of dried fruits into account, they may have the potential of being included into a blood-glucose-managing diet without altering the total carbohydrate intake. The combination of dried fruit and nuts could further ensure a small postprandial glycaemic excursion in a diet and the underlying mechanism of their synergic effects deserves future investigation.

## Figures and Tables

**Table 1:** Composition of test meals (per serving).

Sample	Polished Rice (g)	Dried Fruits (g)	Almonds (g)	Protein (g)	Fat (g)	AC * (g)	Dietary Fibre (g)	Energy (kcal)
Glucose	-	-	-	-	-	50.0	0	200
Rice	66.1	-	-	4.8	0.3	50.0	0.3	222
Ra	-	75.2	-	2.8	0.9	50.0	4.7	219
DApp	-	76.8	-	1.8	0.2	50.0	5.5	209
DJ	-	84.0	-	3.8	0.4	50.0	5.2	219
DApr	-	90.4	-	1.9	0.4	50.0	6.9	211
Ra + R	33.1	37.6	-	3.8	0.5	50.0	3.4	220
DApp + R	33.1	38.4	-	3.3	0.2	50.0	2.8	215
DJ + R	33.1	42.0	-	4.3	0.3	50.0	2.7	220
Apr + R	33.1	45.2	-	3.3	0.3	50.0	3.3	216
A + R	66.1	-	30	8.1	15.3	52.0	5.5	378
Ra + A + R	33.1	37.6	30	9.5	15.6	52.0	8.9	386
DApp + A + R	33.1	38.4	30	9.0	15.3	52.0	9.0	382
DJ + A + R	33.1	42.0	30	10.0	15.4	52.0	8.2	387
DApr + A + R	33.1	45.2	30	9.0	15.4	52.0	8.8	383

Nutritional data were obtained from the nutrition labelling of manufactures. Ra, raisins; DApp, dried apples; DJ, dried jujubes; DApr, dried apricots; Ra + R, mixed meal of raisins and rice; DApp + R, mixed meal of dried apples and rice; DJ + R, mixed meal of dried jujubes and rice; DApr + R, mixed meal of dried apricots and rice; A + R, mixed meal of almonds and rice; Ra + A + R, mixed meal of raisins, almonds and rice; DApp + A + R, mixed meal of dried apples, almonds and rice; DJ + A + R, mixed meal of dried jujubes, almonds and rice; DApr + A + R, mixed meal of dried apricots, almonds and rice. * AC, available carbohydrates.

**Table 2:** Baseline subject characteristics.

Characteristic	Value	
	Mean	SD (SE)
Number of participants (*n*)	11	-
Number of females (*n*)	7	-
Age (year)	21.4	2.4
Body height (cm)	164.0	3.2
Body weight (kg)	55.2	5.2
BMI (kg/m^2^)	20.5	1.4
Fat mass (%)	22.2	2.7
BMR (kcal/d)	1209	58
Fasting plasma glucose (mmol/L)	4.8	0.3

Body composition data are presented as means (SD); plasma glucose data are presented as means (SE).

**Table 3:** Analysis of postprandial glycaemic response characteristics of test samples within 240 min (*n* = 11).

	Test Meal	AUC_0–60_		AUC_60–120_		AUC_0–120_		AUC_120–240_		Peak *		MAGE_0–__240_		NAUC	
		Mean	SE	Mean	SE	Mean	SE	Mean	SE	Mean	SE	Mean	SE	Mean	SE
Reference	Glucose	152.0 ^d^	6.9	81.2 ^ab^	9.2	233.1 ^e^	13.0	10.2 ^a^	1.7	3.9 ^d^	0.1	4.5 ^d^	0.2	−36.7 ^a^	8.8
	Rice	119.7 ^c^	8.3	75.0 ^b^	7.3	194.7 ^d^	14.0	55.2 ^c^	12.5	3.5 ^c^	0.2	3.7 ^c^	0.2	−6.0 ^b^	3.2
Group (1)	DApp	68.4 ^a^	7.6	36.1 ^a^	7.1	104.5 ^a^	12.4	31.2 ^bc^	9.4	2.1 ^a^	0.2	2.2 ^a^	0.2	−4.9 ^b^	2.7
	DJ	87.7 ^ab^	6.8	40.9 ^a^	7.1	129.3 ^ab^	11.5	28.1 ^abc^	8.1	2.6 ^ab^	0.2	2.8 ^b^	0.2	−8.7 ^b^	2.7
	Ra	94.9 ^bc^	9.6	37.8 ^a^	5.6	132.7 ^b^	11.2	19.8 ^ab^	8.6	2.8 ^b^	0.3	3.3 ^bc^	0.3	−26.5 ^ab^	7.3
	DApr	94.1 ^b^	6.3	40.2 ^a^	8.4	134.3 ^bc^	11.4	15.0 ^abc^	7.2	2.7 ^b^	0.2	3.1 ^bc^	0.2	−15.0 ^ab^	5.1
	Glucose	152.0 ^c^	6.9	81.2 ^a^	9.2	233.1 ^c^	13.0	10.2 ^a^	1.7	3.9 ^d^	0.1	4.5 ^d^	0.2	−36.7 ^a^	8.8
	Rice	119.7 ^b^	8.3	75.0 ^b^	7.3	194.7 ^b^	14.0	55.2 ^b^	12.5	3.5 ^c^	0.2	3.7 ^c^	0.2	−6.0 ^b^	3.2
Group (2)	DApp + R	94.2 ^a^	6.0	60.0 ^a^	9.7	154.2 ^a^	12.2	46.2 ^b^	11.2	2.5 ^a^	0.1	2.7 ^a^	0.1	−2.3 ^b^	1.4
	DJ + R	112.6 ^b^	7.3	71.4 ^a^	7.5	184.0 ^b^	12.0	55.6 ^b^	9.6	3.2 ^b^	0.1	3.3 ^b^	0.1	−3.0 ^b^	1.5
	Ra + R	122.9 ^b^	12.5	63.7 ^a^	8.7	186.5 ^ab^	15.2	53.6 ^b^	27.3	3.3 ^bc^	0.3	3.5 ^bc^	0.3	−7.4 ^b^	7.7
	DApr + R	110.6 ^b^	6.3	66.1 ^a^	8.4	176.7 ^ab^	11.4	40.1 ^b^	7.2	3.3 ^bc^	0.2	3.6 ^bc^	0.2	−11.3 ^b^	5.1
	Rice	119.7 ^c^	8.3	75.0 ^d^	7.3	194.7 ^c^	14.0	55.2 ^ab^	12.5	3.5 ^c^	0.2	3.7 ^c^	0.2	−6.0 ^b^	3.2
	A + R	100.4 ^b^	5.0	66.0 ^cd^	5.3	166.4 ^b^	8.9	79.1 ^b^	9.4	2.7 ^a^	0.1	2.8 ^a^	0.1	−1.5 ^a^	1.5
Group (3)	DApp + A + R	89.4 ^ab^	8.1	55.5 ^a^	6.2	144.9 ^ab^	12.1	57.6 ^ab^	8.6	2.6 ^a^	0.2	2.6 ^a^	0.2	−2.9 ^a^	2.3
	DJ + A + R	78.5 ^a^	5.8	49.4 ^abc^	11.7	127.9 ^a^	16.0	45.0 ^a^	12.9	2.5 ^a^	0.2	2.7 ^a^	0.2	−5.0 ^a^	2.6
	Ra + A + R	92.9 ^b^	7.1	37.8 ^b^	5.5	130.7 ^a^	10.3	38.0 ^a^	6.8	2.8 ^a^	0.2	2.9 ^a^	0.2	−4.3 ^a^	1.8
	DApr + A + R	94.0 ^b^	5.5	58.6 ^acd^	6.2	152.7 ^ab^	9.3	47.5 ^a^	8.1	2.8 ^a^	0.2	2.9 ^a^	0.2	−5.1 ^a^	3.3

Ra, raisins; DApp, dried apples; DJ, dried jujubes; DApr, dried apricots, Ra + R, mixed meal of raisins and rice; DApp + R, mixed meal of dried apples and rice; DJ + R, mixed meal of dried jujubes and rice; DApr + R, mixed meal of dried apricots and rice; A + R, mixed meal of almonds and rice; Ra + A + R, mixed meal of raisin, almonds and rice; DApp + A + R, mixed meal of almonds, dried apples and rice; DJ + A + R, mixed meal of dried jujubes, almonds and rice; DApr + A + R, mixed meal of dried apricots, almonds and rice. * The incremental peak of blood glucose value. ^a,b,c,d,e^ Mean values within a column with unlike superscript letters are significantly different (*p* < 0.05).

**Table 4:** Glycaemic indices (GIs) of test samples (*n* = 11).

Test Group	Ra		DApp		DJ		DApr		A + R		Rice		Glucose
	Mean	SE	Mean	SE	Mean	SE	Mean	SE	Mean	SE	Mean	SE	
Dried fruits	56 ^abA^	5	43 ^abA^	4	55 ^abA^	6	56 ^abA^	4	-	-	81 ^dB^	4	100 ^e^
Dried fruits + R	77 ^abB^	8	65 ^abB^	5	77 ^abB^	6	75 ^abB^	7	-	-	81 ^bB^	4	100 ^c^
Dried fruits + A + R	54 ^abA^	2	60 ^aA^	4	52 ^aA^	4	64 ^cdB^	4	70 ^d^	4	81 ^eA^	4	100 ^f^

Ra, raisins; DApp, dried apples; DJ, dried jujubes; DApr, dried apricots; A + R, mixed meal of almonds and rice. ^a,b,c,d,e,f^ Mean values within a row with unlike superscript letters are significantly different (*p* < 0.05). ^A,B^ Mean values within a column with unlike superscript letters are significantly different (*p* < 0.05).

**Table 5:** Sugar profiles and other components of dried fruits used in test meals.

Nutrient	Dried Apples		Dried Jujubes		Raisins		Dried Apricots	
	Mean	SD	Mean	SD	Mean	SD	Mean	SD
Sucrose ^§^ (g/100 g)	11.7 ^b^	0.3	30.1 ^c^	0.6	0.1 ^a^	0.0	30.1 ^c^	0.6
Glucose ^§^ (g/100 g)	10.1 ^a^	0.2	13.6 ^b^	0.3	27.7 ^c^	0.4	15.8 ^b^	0.3
Fructose ^§^ (g/100 g)	39.0 ^d^	0.6	19.1 ^b^	0.3	30.0 ^c^	0.6	8.1 ^a^	0.1
Total acid (g/100 g)	1.30 ^b^	0.04	1.04 ^a^	0.04	1.16 ^ab^	0.03	1.74 ^c^	0.03
Pectin (g/100 g)	1.17 ^b^	0.05	1.36 ^c^	0.03	0.47 ^a^	0.01	1.93 ^d^	0.03
Dietary fibre (g/100 g)	8.7 ^c^	0.3	6.2 ^a^	0.2	8.8 ^c^	0.2	7.0 ^b^	0.2
Total polyphenols (mg GAE/100 g)	389.8 ^b^	9.3	457.4 ^c^	10.1	683.6 ^d^	13.0	151.2 ^a^	7.4
Oxygen radical absorbing capacity (mmol Trolox/100 g)	26.0 ^c^	1.2	9.4 ^a^	0.7	30.4 ^d^	1.1	13.1 ^b^	0.5

^§^ Recovery ≥95%; ^a,b,c,d^ Mean values within a row with unlike superscript letters are significantly different (*p* < 0.05).

**Table 6:** The percentages of soluble sugar.

Soluble Sugar	Dried Apples	Dried Jujubes	Raisins	Dried Apricots
Sucrose * (%)	19.2	47.9	0.2	55.7
Glucose * (%)	16.6	21.7	47.9	29.3
Fructose * (%)	64.1	30.4	51.9	15.0

* The figures were represented as the percentage in total sugar.

**Table 7:** Correlation between sugar contents and postprandial GR characteristics of dried fruit-containing meals.

Sugar	AUC_0–60_	AUC_60–120_	AUC_0–120_	AUC_120–240_	Peak	MAGE_0–240_	NAUC
	(mmol/L·h)	(mmol/L·h)	(mmol/L·2 h)	(mmol/L·2 h)	(mmol/L)	(mmol/L)	(mmol/L·4 h)
Fructose	0.871	0.210	0.747	0.910	0.943	0.928	0.478
Glucose	0.287	0.982	0.380	0.423	0.237	0.165	0.015
Sucrose	0.298	0.110	0.232	0.311	0.369	0.440	0.864
Total fructose ^a^	0.070	0.329	0.073	0.058	0.115	0.116	0.376
Total glucose ^b^	0.953 *	0.850	0.985 *	−0.756	0.931	0.871	0.953 *
Total fructose/total glucose	−0.978 *	−0.791	−0.995 **	0.807	−0.957 *	−0.915	0.624

* *p* < 0.05, ** *p* < 0.01. ^a^ The combination of free fructose and the fructose unit in sucrose. ^b^ The combination of free glucose and the glucose unit in sucrose.
